# Fully Automated Characterization of Protein–Peptide Binding by Microfluidic 2D NMR

**DOI:** 10.1021/jacs.2c13052

**Published:** 2023-01-30

**Authors:** Marek Plata, Manvendra Sharma, Marcel Utz, Jörn M. Werner

**Affiliations:** † School of Chemistry, University of Southampton, SouthamptonSO17 1BJ, United Kingdom; ‡ School for Biological Sciences, University of Southampton, B85 Life Science Building, University Rd, SouthamptonSO17 1BJ, United Kingdom

## Abstract

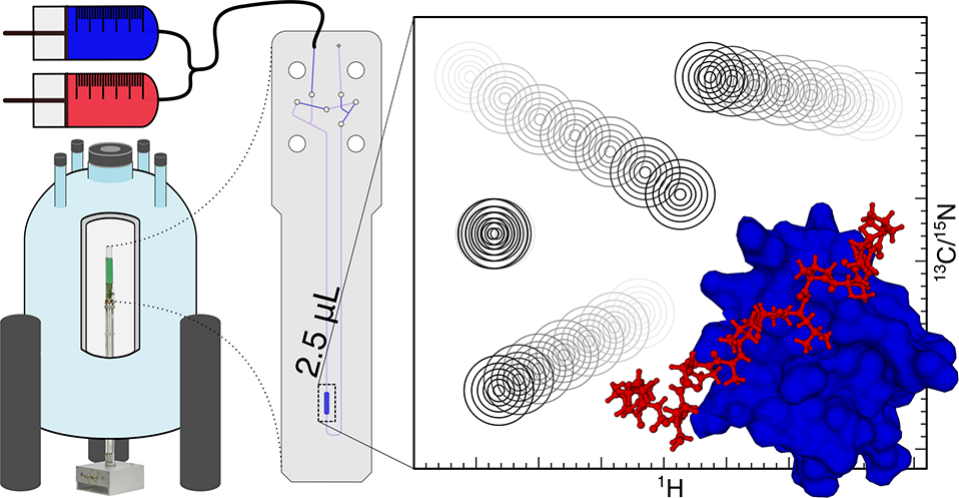

We demonstrate an automated microfluidic nuclear magnetic resonance (NMR) system that quantitatively characterizes protein–ligand interactions without user intervention and with minimal sample needs through protein-detected heteronuclear 2D NMR spectroscopy. Quantitation of protein–ligand interactions is of fundamental importance to the understanding of signaling and other life processes. As is well-known, NMR provides rich information both on the thermodynamics of binding and on the binding site. However, the required titrations are laborious and tend to require large amounts of sample, which are not always available. The present work shows how the analytical power of NMR detection can be brought in line with the trend of miniaturization and automation in life science workflows.

## Introduction

Microfluidic lab-on-a-chip (LoC) devices provide unique, convenient, and reproducible platforms for the interrogation of complex biological systems under highly controlled conditions. They employ a wide range of readout methods to quantify system responses to defined stimuli.^1,2^ Accordingly, they have the potential to transform experimentation in the life sciences.^3^ Limited sample use and integration of complex functionalities in LoC devices are intrinsically suited to the miniaturization and parallelization of biomedical workflows.^4^ Biological systems at all scales, ranging from whole organisms down to subcellular organelles and molecular assemblies, have been studied in this way.^5−9^ However, studies of protein–ligand interactions have so far relied on readout methods which require ligand modifications and lack detailed information on the molecular scale.^10^

Nuclear magnetic resonance (NMR) is uniquely placed for characterizing macromolecular systems, including protein–ligand interactions. It has the ability to determine the number and location of interaction sites, allosteric effects, and the atomic structures and dynamics of ligands as well as protein and to evaluate the thermodynamics of the interaction.^11,12^ NMR offers unique tools for structural and molecular biologists as well as medicinal chemists, because it does not rely on any protein or ligand modification for detection and offers substantial freedom in the choice and variation of buffer conditions. In addition, NMR is capable of probing a broad range of affinities (nM–mM).^13^ In a standard modality of protein-detected ligand interactions, a series of 2D heteronuclear spectra of a uniformly labeled protein (either ^15^N or ^13^C) are performed with increasing ligand-to-protein molar ratios ([L]/[P]). The fraction of protein bound to the ligand is then reflected in chemical shift perturbations (CSPs), Δδ, of the resonances in the protein NMR spectrum.^12,14^ Once the resonances are assigned to the nuclei under investigation, the CSPs can be used to determine the ligand binding site. Analysis of CSPs of the backbone amides using ^1^H–^15^N HSQC experiments takes advantage of the fact that amides provide a single probe at every backbone position of a protein, with exception of prolines, but are typically limited to proteins below about 100 kDa;^15−17^ much larger proteins or molecular assemblies and even MDa protein complexes are experimentally accessible by detecting methyl groups.^18−20^

Conventional NMR implementation of binding experiments in a standard 5 mm probe requires approximately 600 μL liquid samples and a significant amount of isotope-labeled protein and involves repeated manipulation by the experimenter. It is known that the signal-to-noise (SNR) of small NMR detectors scales favorably with size. A number of authors have presented miniaturized NMR detection systems that benefit from this effect.^21−23^ Furthermore, recent progress in the integration of micro-NMR detectors with complex microfluidic devices are enabling automation of experimental protocols in situ.^24−27^ In the following, we demonstrate the automation of NMR binding experiments by coupling microfluidic control of sample mixing with NMR detection in a sample volume of 2.5 μL.

## Microfluidic NMR Titration Setup

**Figure 1:**
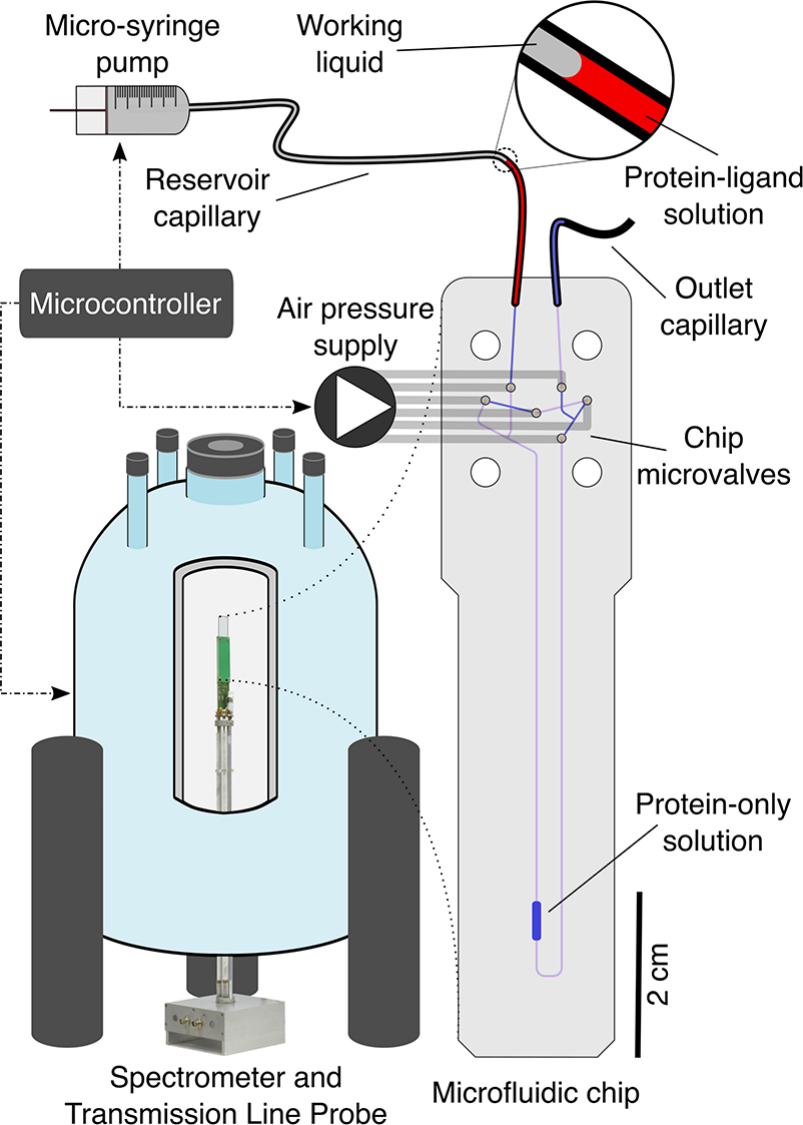
Schematic representation of the device and its operation inside the NMR spectrometer after the initial filling procedure carried out by the experimenter.

**Figure 2:**
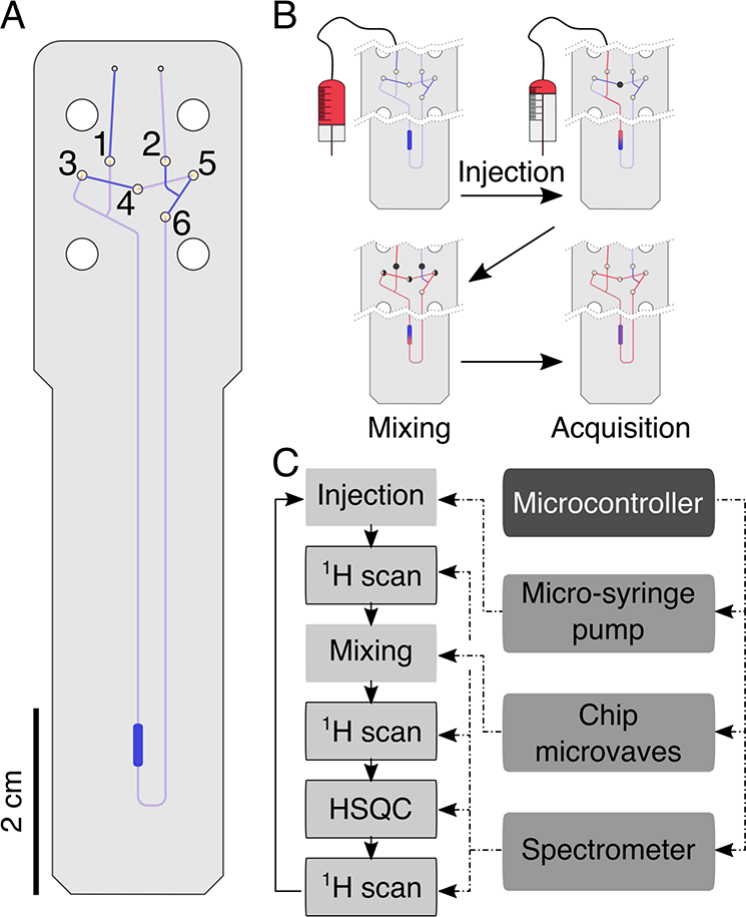
Detailed representation of (A) the microfluidic chip and (B) the device operation during the automated titration experiment. Filled black circles over the chip microvalves in (B) indicate a closed valve, while half-filled circles signify the periodic valve actuation for peristaltic mixing. In (B) a single block of the automated experiment is detailed, highlighting the hardware elements influenced by the microcontroller for each step.

The automated microfluidic NMR titration system consists of several hardware components whose actions are coordinated by a microcontroller, as indicated in Figure 1. The transmission line probe is optimized for double-resonance (^1^H–^15^N or ^1^H–^13^C) protein detection and is specifically designed to accept planar microfluidic devices, ensuring considerable freedom in the design of the microfluidic chip.^25,28^ The microfluidic chip houses six pneumatic on-chip valves^24^ that are used to control the movement of liquid on the chip and a 2.5 μL detection chamber that is positioned in the volume of maximal probe sensitivity. The upper part of the chip is encapsulated by a pair of 3D-printed holders that provide interfaces to the fluidic and pneumatic infrastructure outside of the NMR magnet (cf. the Supporting Information). Chip access valves (valves 1 and 2 in Figure 2A) act to isolate the liquid circuit (∼10 μL) on the microfluidic chip from the external supply. Three valves on the bridge pathway (valves 3–5 in Figure 2A) can be actuated periodically for peristaltic circulation of the liquid in the chip. This mechanism is used for mixing of the solutions inside the microfluidic chip circuit.^24,29,30^ The titration experiment requires (a) a protein-only solution and (b) a solution with the same protein concentration and a molar excess of ligand sufficient to saturate binding. Both solutions are prepared in the same buffer and include trimethylsilylpropanoate (TSP) as a chemical shift standard. The concentrations of TSP in the two solutions differ by at least 1 order of magnitude so that the TSP resonance intensity in the NMR spectrum can be used as an internal standard for the mixing ratio of the two solutions. In the preparation of the experiment, 15 μL of solution b, followed by 15 μL of solution a, is pulled into the reservoir capillary. The capillary is then connected to the chip, and the experiment is started. Liquid injection of the solutions by the microsyringe pump and microvalve actuation, necessary for opening and sealing the access to the chip and peristaltic mixing, is coordinated by the microcontroller, which also triggers the NMR acquisition.

To start the titration experiment, 10 μL of the protein-only solution (a) is injected into the chip using the microsyringe while the middle valve of the bridge pathway (valve 4, Figure 2A) is closed. Afterward, the ^1^H and 2D HSQC spectra specified in the titration schedule are acquired. In each subsequent experiment a 2 ± 0.25 μL volume of the protein–ligand solution b is injected into the mixing circuit, mixed with the previously analyzed solution, and further spectra are acquired. The titration schedule, detailing the acquisition and automation steps, is shown in Figure 2B,C. ^1^H spectra are used as an internal standard through the linear relationship between the intensity of the TSP signal and the ligand concentration (see eq 4 below). This fully automated process delivers a series of 2D HSQC spectra as a function of the ligand to protein ratio, from which the binding process can be quantified.

## Results and Discussion

**Figure 3:**
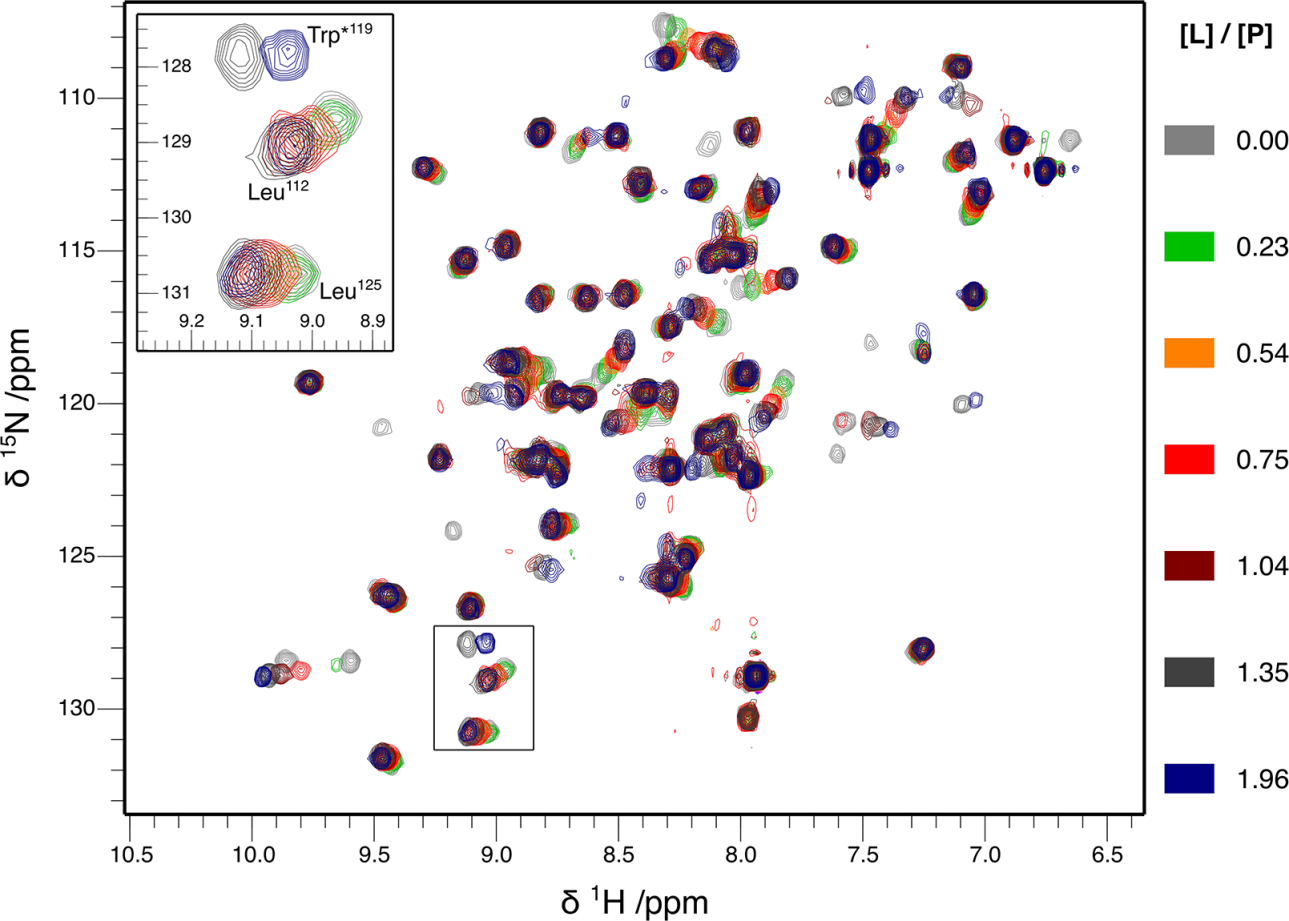
Overlay of selected ^1^H–^15^N HSQC spectra of hFynSH3, obtained during the automated titration experiment with p85α_P91-T104_. The coloring scheme represents the increasing concentration of p85α_P91-T104_ as indicated on the right. For clarity, only seven ^1^H–^15^N HSQC spectra are shown, at specified molar ratios of ligand to protein. The starred Trp*^119^ label refers to a signal originating from the tryptophan side chain NHε.

The capabilities of this microfluidic system are demonstrated using the well-characterized interaction of the SH3 domain of the human Fyn protein hFynSH3 with the 13 amino acid peptide fragment of the p85α subunit of PI 3-kinase, p85α_P91-T104_.^31,32^ Here, we focus on the NH resonances of hFynSH3 using ^1^H–^15^N HSQC experiments, while the feasibility of methyl detection is demonstrated in the Supporting Information. Figure 3 shows an overlay of the hFynSH3 spectra, acquired at increasing p85α_P91-T104_ concentrations. The signals of each nucleus in the titration series follow a straight line in response to increasing [L]/[P], implying that the analysis of the binding equilibrium requires only two states, i.e. free and ligand-bound protein, and that the CSPs represent the fraction of the bound protein.^12^ In each spectrum presented in Figure 3 61 μg of protein was present in the 2.5 μL detection volume, resulting in the SNR of 32:1 and an apparent resolution below 10 Hz (a more detailed discussion of the probe performance and spectral quality is provided in section 2 of the Supporting Information). Using a standard HSQC sequence,^33^ the entire titration series of 11 experiments was completed in approximately 24 h. The SNR achieved for the ^1^H–^15^N HSQC experiment has allowed us to unambiguously assign 90% of the observable amide signals of hFynSH3.

**Figure 4:**
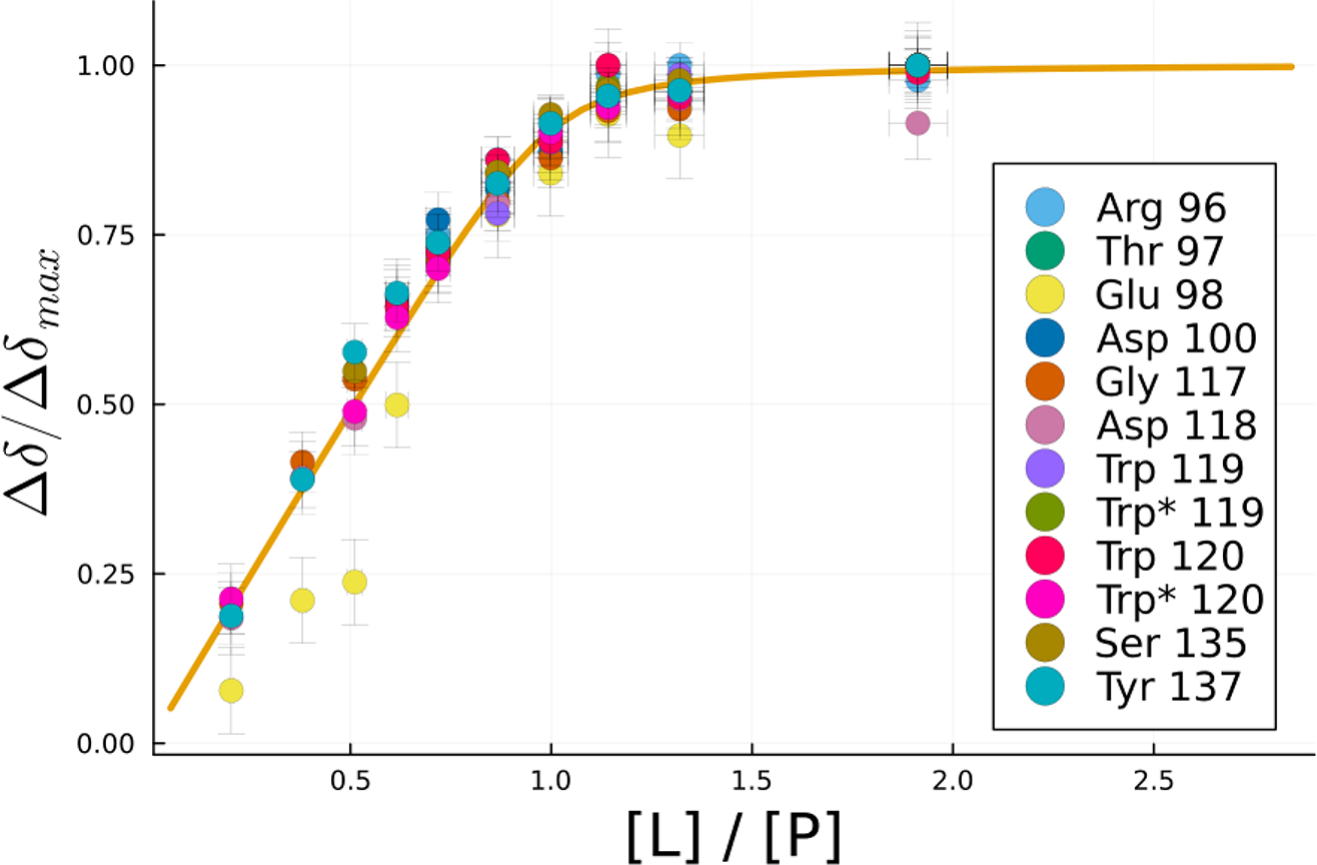
Binding isotherm for the amide hFynSH3 signals above the σ_c_ significance threshold. The data points are the fractional shift values for individual signals. The solid line represents the best fit for the entire data set, with the exception of Glu^98^, which is affected by spectral overlap with Tyr^132^. Trp* labels refer to signals originating from the tryptophan side chain NHε groups.

The ligand titration was carried out to the point where the protein was saturated with the ligand, as assessed by minimal changes in CSPs in the last two titration spectra. All isotope-weighted differences between the free form hFynSH3 and fully saturated protein signals, Δδ_max_, were used to evaluate the corrected standard deviation, σ_c_,^34^ as the cutoff value over which the significant CSPs were selected. The data points in Figure 4 represent the combined ^1^H and ^15^N fractional shifts (Δδ/Δδ_max_) for the significant hFynSH3 amides plotted against [L]/[P] as determined from the concentration of TSP in the analyzed solution.

In order to obtain the binding constant *K*_D_, the standard equation describing the fractional chemical shift as a function of protein and ligand concentration and *K*_D_(12) was recast using a normalized ligand concentration, α, and dissociation constant, β_D_, defined as
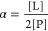
1
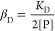
2where [L] denotes the total concentration of ligand and [P] is the total concentration of protein. This yields an expression for the fractional chemical shift with dimensionless parameters, which facilitates separating the contributions of different errors:
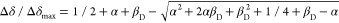
3While in conventional experiments the ligand concentration depends on manual mixing of samples, in the present case the ligand concentration is automatically calculated from the signal intensity of the TSP resonance at 0 ppm, *I*_TSP_. The normalized ligand concentration, α, at any titration step is linearly dependent on the measured *I*_TSP_ as
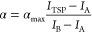
4where *I*_A_ and *I*_B_ are the intensities of the TSP signals in the ligand-free and in the ligand-saturated solutions, respectively, and α_max_ is the normalized ligand concentration in the ligand-saturated solution. The resulting collective fit of all CSP data above the significance threshold is shown as a solid line in Figure 4. This yields *K*_D_ = 48 ± 9 μM, in excellent agreement with the previously published value of 50 μM.^31^

**Figure 5:**
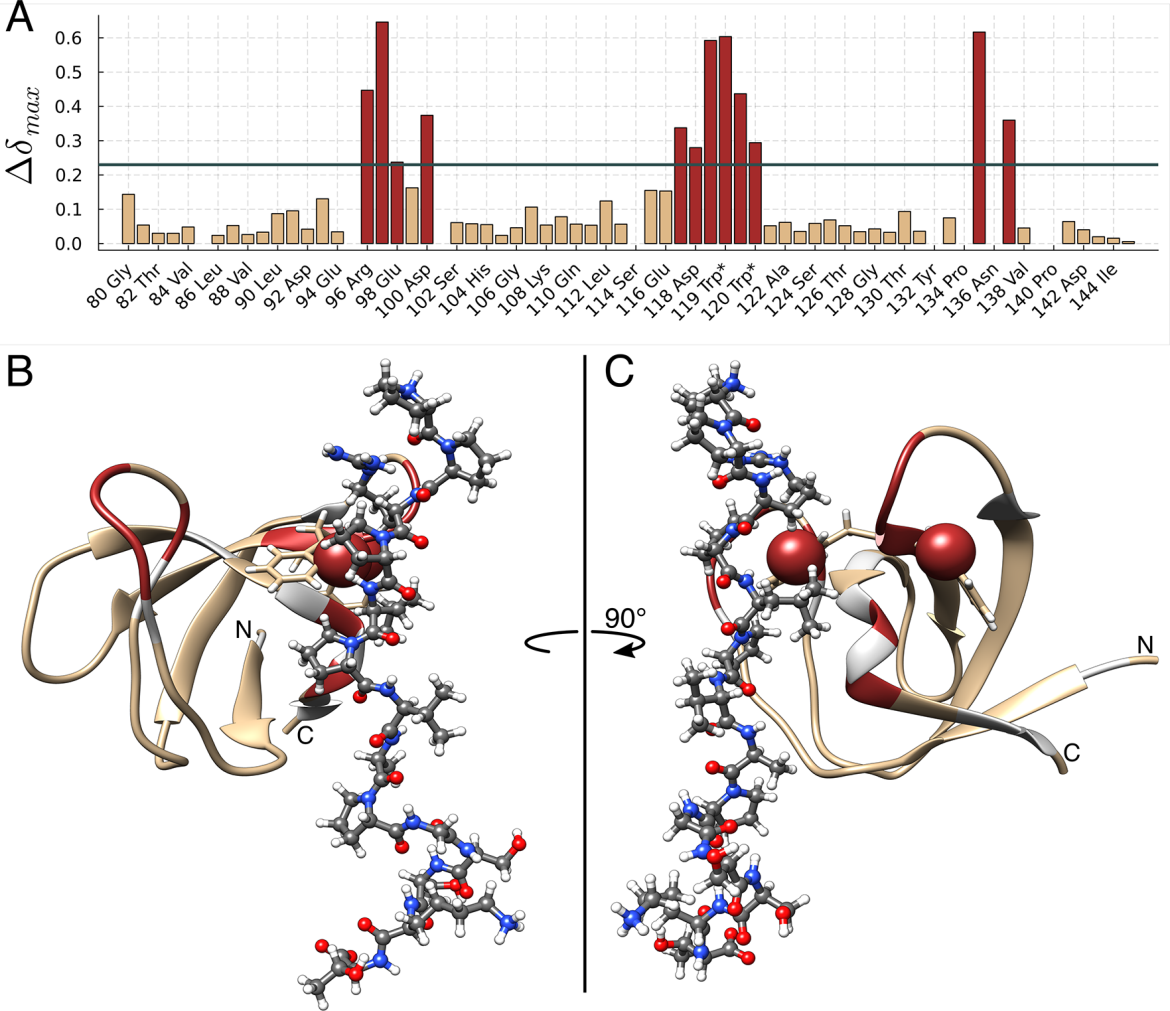
Definition of the binding site for p85α_P91-T104_ on hFynSH3. In (A) a histogram of maximum chemical shift perturbations, Δδ_max_, is plotted as a function of the amino acid sequence of hFynSH3. Trp* labels refer to the Δδ_max_ reported by the side chain NHε of tryptophan residues, and the horizontal line represents the significance threshold, σ_c_. In (B) and (C) Δδ_max_ values are mapped on the backbone structure of hFynSH3 (PDB entry: 1AZG).^32^ Colored in brown are the Δδ_max_ values above σ_c_, including the tryptophan NHε shown as spheres, in beige are the Δδ_max_ below σ_c_, and unassigned amides are shown in light gray. The p85α_P91-T104_ peptide is shown in ball and stick representation using the standard atom color coding.

The binding site was identified from the fully saturated chemical shifts Δδ_max_. In Figure 5 the Δδ_max_ distribution is shown with respect to the known hFynSH3 sequence.^31,32^ From a total of 59 assigned amide signals, 12 show perturbations above the significance threshold. They map onto three separate patches between Arg^96^-Asp^100^, Gly^117^-Trp^120^, and Tyr^135^-Tyr^137^ on the hFynSH3 backbone, which is consistent with previously published data.^31^

## Conclusions

In summary, we have successfully automated protein–peptide binding analyses using microfluidic NMR. The results clearly demonstrate the potential of microfluidic NMR for automation of complex analytical protocols, which would otherwise be laborious and time-consuming to perform. This aligns well with a general trend in the life sciences toward miniaturization and automation. The current platform that is working with microliter volumes brings the analytical power of NMR in line with microliter protein protein expression systems^35,36^ for generating fully automated discovery workflows. Further improvements could be realized by combining recent advances in autonomous processing of protein NMR data.^37−45^ The modularity of the system and flexibility of the microfluidic chip design is such that the device can easily be adapted to suit specific experimental needs such as screening applications, protein–protein interactions and studies of multiligand equilibria. In addition, the device could be used for deuterium exchange experiments and protein-folding studies in response to buffer changes. A particularly exciting prospect is the integration of hyperpolarized binding studies^46^ in a microfluidic platform.^47^ The experiment only uses a modest amount of protein, about 0.7 mg in the current implementation. There is significant scope for further reduction of the total amount of protein and the protein concentration used for the experiment. In particular, the current chip design has a working volume of about 10 μL, but only a 2.5 μL detection volume. This ratio could be brought close to 1:1 by increasing the detection volume or reducing the cross-section of the transport channels and improved further by changing the fabrication technology to achieve smaller tolerances. The current design discards a part of the protein/ligand solution at every step of the experiment, as it is displaced out of the chip by the injected increment. This mode of operation was chosen to keep the chip design simple. An implementation that operates with a closed cycle and takes an NMR spectrum of a large fraction of the available protein at every step is feasible. Efforts in this direction are underway in our laboratory and will be reported on at a later occasion.

## Materials and Methods

### Sample Preparation

Expression and purification of ^13^C- and ^15^N-labeled protein samples was carried out according to the previously published protocol for hFynSH3^31^ (sequence numbering according to UniProtKB entry: P06241 FYN_HUMAN). Protein concentrations in the titration samples were evaluated based on the light absorbance measurements at 280 nm using the molar extinction coefficient ε_280_ = 16960 M^–1^ cm^–1^.

The p85α_P91-T104_ peptide was obtained as >97% purity lyophilized powder from the supplier (ChinaPeptides, CN). Samples for analysis by NMR were prepared by dissolving the powder in 1 mL of H_2_O and twice dialyzing in a 1/1000 volume ratio using the 0.5 MWCO Float-A-Lyzer G2 device (Repligen, US). Following dialysis, the 1 mL sample was lyophilized for storage and dissolved in a designated volume of the buffer solution in preparation for the NMR experiments. The peptide concentration in the protein–ligand sample was calculated from the intensity ratios of TSP to Leu^95^ and Val^97^ methyl signals of the p85α_P91-T104_ peptide in ^1^H NMR, while ensuring the interscan delay exceeded 5× the proton *T*_1_ values.

### Microfluidic Chip Fabrication

Microfluidic devices were made by laser cutting and thermal bonding of poly(methyl methacrylate) sheet material as previously described.^48^

### Experimental Automation

Experiments were automated using an external precision microsyringe pump (LabSmith, California, USA) and a home-built microcontroller system as described above. The full details of the design and operation of the system are provided in section 1 of the Supporting Information.

### NMR Acquisition and Data Processing

All NMR measurements were performed at 14.1 T in a Bruker AS 600 MHz WB magnet equipped with a Bruker AVANCE NEO console, using a modular transmission-line microfluidic NMR probe following a previously published design, which was adapted to ^15^N spectroscopy as detailed in the Supporting Information. For each ^1^H spectrum 16 transients were recorded with a repetition delay of 4 s. Water signals were suppressed by 4 s of presaturation with a nutation frequency of 83 Hz. All ^1^H processing was done using the NMR.jl (https://github.com/marcel-utz/NMR.jl) package for the Julia programming language,^49^ implementing 1 Hz of Lorentzian line broadening and automatic baseline correction. HSQC spectra were recorded with a 1.0 s repetition delay; direct and indirect acquisition times were 92 and 16 ms, respectively. All HSQC spectra were processed using the NMRPipe software^50^ implementing cosine-bell apodization, linear prediction, and zero filling to double the number of data points.

CSPs were obtained from the ^1^H and ^15^N chemical shift differences^12^
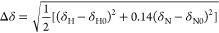
5where δ_H0_ and δ_N0_ are the proton and nitrogen chemical shifts of the free protein, respectively. The data fitting was done by the Levenberg–Marquardt least-squares algorithm as implemented in the LsqFit package of the Julia programming language. CSPs above the significance threshold were fitted as a function of the intensity of the TSP signal with the binding constant *K*_D_ as well as the slope and intercept of the linear relationship between the normalized ligand concentration and the TSP signal intensity as free fit parameters. Errors of *K*_D_ are reported as 95% confidence intervals.^51^

## Supporting Information Available

Complete technical description of the apparatus and automation protocol, improvements made to the microfluidic NMR probe, discussion of the quality of the ^1^H–^15^N and ^1^H–^13^C HSQC spectra, and a more detailed description of the data fitting protocol (PDF)
