# Supplementary material for: Fully Automated Characterization of Protein–Peptide Binding by Microfluidic 2D NMR
Source: J Am Chem Soc. 2023 Jan 30;145(5):3204–10. doi: 10.1021/jacs.2c13052 (PMC9912330; doi:10.1021/jacs.2c13052)
Supplement: Supplementary file 1 — ja2c13052_si_001.pdf [file ja2c13052_si_001.pdf]

Supplementary information to: Fully automated characterization of protein-peptide binding by microfluidic 2D NMR

Marek Plata, Manvendra Sharma, Marcel Utz and Jörn M. Werner

Contents

1 Details of the apparatus **1**
1.1 Microfluidic Device . . . 1
1.2 Automation . . . 3
1.3 Improved Transmission-Line Probe . . . 4

2 Quality and Characteristics of microfluidic HSQC spectra **5**

3 K_D Fitting **6**

1 Details of the apparatus

1.1 Microfluidic Device

Described here is the automated microfluidic system for characterisation of protein-ligand interactions by high-resolution heteronuclear NMR. The following expands on the technical details of this setup and its operation as well as supplementary results.

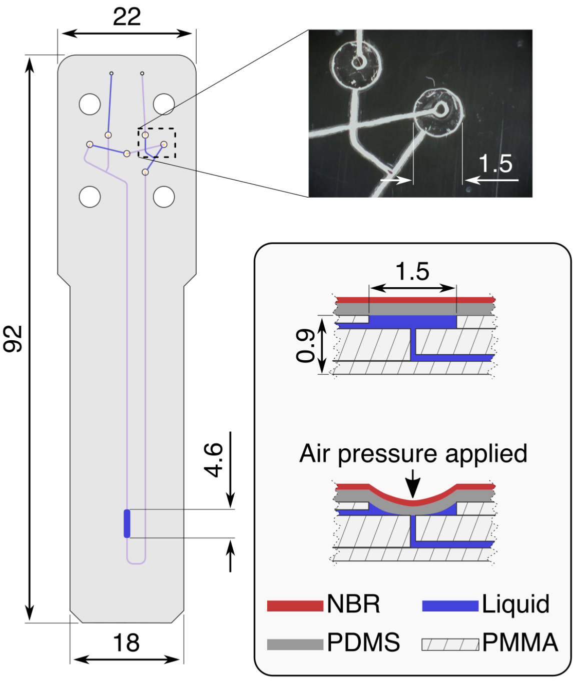

Figure S1: Microfluidic chip design and the microvalve architecture. In the main frame the full chip is shown with the micrograph blow-out of the chip microvalves in the top right. In the bottom right panel, the cross section of an individual valve is shown including the actuation principle. All dimensions are in mm units.

The overall design of the microfluidic device was described previously[1], including the ancillary hardware and interfaces. Here, we provide a concise description of the operation of the system for the reader's convenience, along with some additional technical details. This platform relies on the design versatility of the transmission-line probe (TLP), which accommodates planar microfluidic devices inside a standard NMR spectrometer. The probe and device are of corresponding geometry, such that the 2.5 μL detection chamber on the microfluidic chip coincides with the area of maximum sensitivity of the TLP.[2] The chips are fabricated from three layers of polymethyl methacrylate (PMMA) with laser-cut outline and channels, bonded under heat and pressure.[3] The key element of the chip design is a set of pneumatic microvalves, positioned away from the detection area as shown in S1. For full functionality the chip is complemented with two holders with interfaces to the hardware that operates outside of the spectrometer: a micro-syringe to inject the sample liquid, and pressurized air supply to actuate the microvalves. The chip is sandwiched by the the holders with additional elastomer membranes in between, as shown in S2. Polydimethylsiloxane (PDMS) acts as a gasket and provides a seal for the liquid, while nitrile rubber (NBR) acts as gas barrier, preventing the pressurized air permeating into the sample liquid. The microvalve function relies on the deformation of the elastomer membranes, which are pushed by ~ 5 bar pressurized air against the valve floor. When actuated sequentially, they generate a peristaltic flow within a closed circuit on the chip, mixing the liquids inside. The temporal delivery of pressurized air to the microvalves is controlled by a set of solenoid valves connected to the air supply outside of the spectrometer.

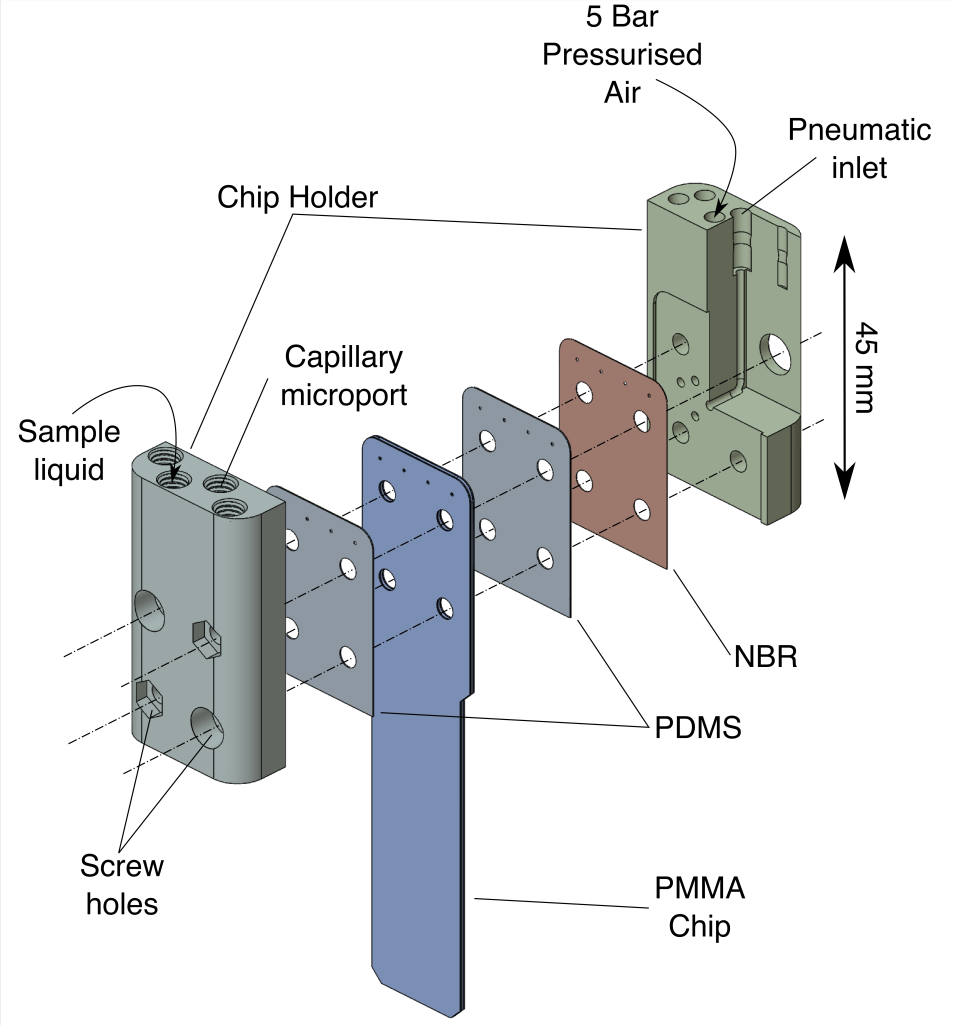

Figure S2: Microfluidic device assembly from all components.

Previously stated mixing efficiency[1] was established by observations of a color change after injection of a colored dye into the aqueous solution present inside the chip sample chamber. In preparation for the current work analogous experiments were carried out inside the spectrometer, where an aqueous solution of DSS and Fumaric Acid was gradually injected into a Fumaric Acid only solution, and the changes of the NMR signals

of DSS and Fumaric acid were quantified. Mixing efficiency was evaluated by observing the change in the intensity of the 0 ppm signal for DSS after a given mixing period, following the initial injection into the detection chamber. In Fig. S3 the normalized intensities of the DSS and Fumaric Acid signals are plotted against the cumulative mixing time. While the initial signals are of similar intensity, after mixing the intensity of the DSS signal drops, while the Fumaric Acid signal remains essentially unchanged. Decreasing DSS signal intensity implies the detected concentration of the compound is lower, as it is being more evenly distributed within the circuit of the microfluidic chip, as result of the peristaltic mixing. Most of the DSS signal disappears after 4 minutes, which was the previously established mixing time, however the signal flattens out only after 8 - 12 minutes of mixing. As a result, the mixing period was conservatively extended to 12 minutes for all experiments discussed in this work.

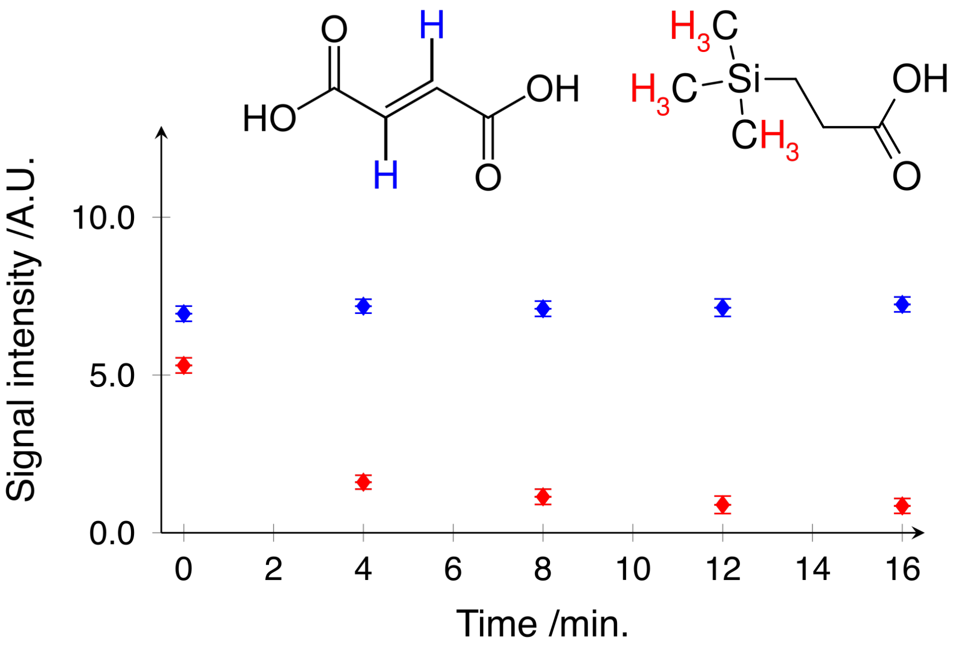

Figure S3: Evaluation of the mixing efficiency for the microfluidic device. The plotted data show the intensity values for the 0 ppm and 6.52 ppm peaks originating from DSS and Fumaric Acid, respectively. Data were acquired by NMR after initial injection of DSS and Fumaric Acid solution into the Fumaric Acid only solution (time point 0), and following specified mixing intervals.

The experimental protocol was structured to minimize intervention by the operator. In preparation, 15 μL each of the protein-ligand and protein-only samples were consecutively loaded into the reservoir capillary extending from the micro-syringe to the microfluidic device, before connecting to the device inlet. The outgoing outlet capillary was connected to 1.5-3 bar back pressure to maintain the metering accuracy inside the microfluidic chip equal to the movement of the micro-syringe plunger. An initial push of 10 μL from the micro-syringe filled the chip circuit with the protein-only solution, and the device was placed inside the spectrometer. The following steps were executed automatically as specified in the experimental schedule. Each step included a pre-specified volume push from the micro-syringe that injected a fraction of the protein-ligand solution into the chip, and subsequently the two solutions were mixed as result of a peristaltic motion induced by sequential actuation of the microvalves. After each injection and mixing, a ^{1}H spectrum was recorded in order to measure the intensity of the 0 ppm signal of TSP, an internal standard present in known molar excess in the protein-ligand solution, therefore quantification the ligand concentration in the sample chamber was closely monitored. Following the mixing and acquisition of the ^{1}H spectra, the HSQC spectrum was recorded before a new injection step was implemented.

1.2 Automation

System control software was designed in LabView to control the output from all ancillary hardware, the six solenoid valves (Festo, DE) actuated by the Arduino Mega 2560 (Arduino, US) controller and the micro-

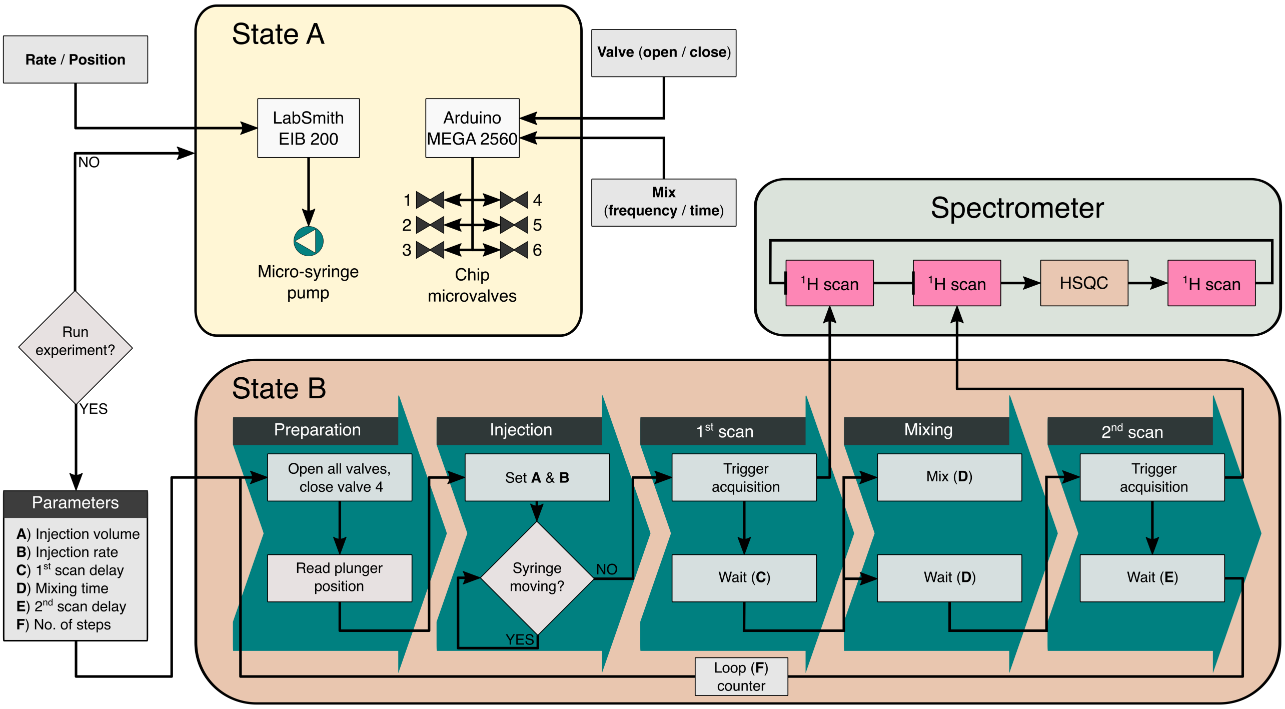

Figure S4: Flow diagram for the automated titration experiment. The control software can operate in a manual (A) or automated (B) state. In the manual state the operator has full control over the parameters that control the micro-syringe pump and chip microvalves. In the automated state the user needs to specify the six experimental parameters before the the pre-programmed sequence is executed. The experiment is carried out until all specified steps are finished. If needed, the operator can also abort the experiment at any time.

syringe pump (SPS01; LabSmith, US). The software operates in two distinct states: **(A)** where all commands to the individual components are input manually and **(B)** where the titration experiment is executed automatically, according to the specified schedule. Spectrometer operates separately, carrying out acquisition according to the specified schedule. In each titration step four spectra are acquired, after each injection and mixing the spectrometer awaits the 5 V signal sent from the control software to initiate acquisition.

1.3 Improved Transmission-Line Probe

Initial feasibility of the concept was proved using an existing modular TLP probe, described in detail by Sharma and Utz[2]. This probe uses a circuit in which the two detector planes are connected at the far end through a pair of capacitors. This design leads to a relatively low inductance of the detector assembly at low frequencies, which makes it difficult to tune and match the system to the ^{15}N frequency. As a result, the probe initially available was only tunable to ^{13}C. A series of ^{1}H-^{13}C HSQC spectra obtained with this probe are shown in Fig. S6. While these spectra do show systematic shifts of some of the methyl resonances which could be interpreted, the quality in terms of SNR and the magnitude of the shifts compared to the line width was not sufficient for a reliable fit of the binding constant, as discussed in section 2. We therefore set out to build a variant of the probe designed for efficient acquisition of ^{1}H-^{15}N HSQC spectra with optimal sensitivity.

Efficient coupling to the low Larmor frequency of ^{15}N at 14.7 T (600 MHz) is facilitated by a high inductance of the detector coil. The two conductor planes that make up the transmission line detector were therefore directly connected at the top by replacing the capacitive connection with conducting blocks of copper, as shown in Fig. S5. In addition, the tank circuit band reject filter of the original TLP design that insulates the ^{15}N channel from the ^{1}H frequency was replaced by a shorted $\lambda/4$ line, as shown in the figure. This probe was found to perform very well, with a radiofrequency efficiency of 26 and 82 μT/$\sqrt{W}$ for the ^{1}H and ^{15}N channels, yielding 90° pulse lengths of 3.2 μs and 10 μs, respectively. A full description of this probe with a complete characterization of its performance will be reported in a separate paper.

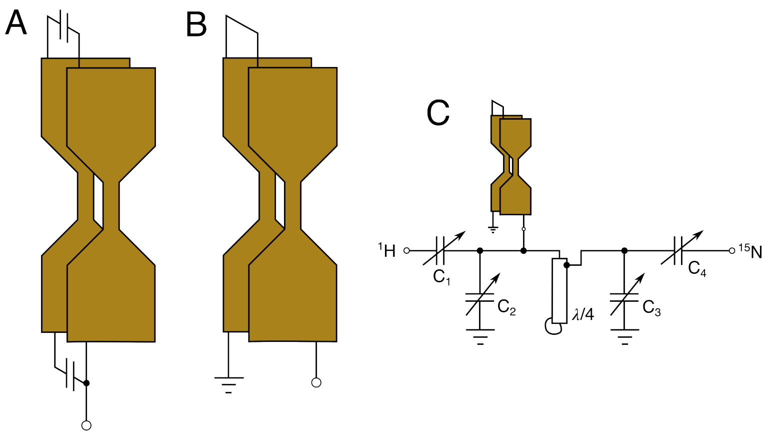

Figure S5: A: Original transmission line resonator as described by Sharma and Utz [2] with capacitive coupling of the detector planes; B: improved detector with increased inductance due to direct coupling of the detector planes at the top; C: circuit diagram of the improved ^{1}H-^{15}N double resonance probe.

2 Quality and Characteristics of microfluidic HSQC spectra

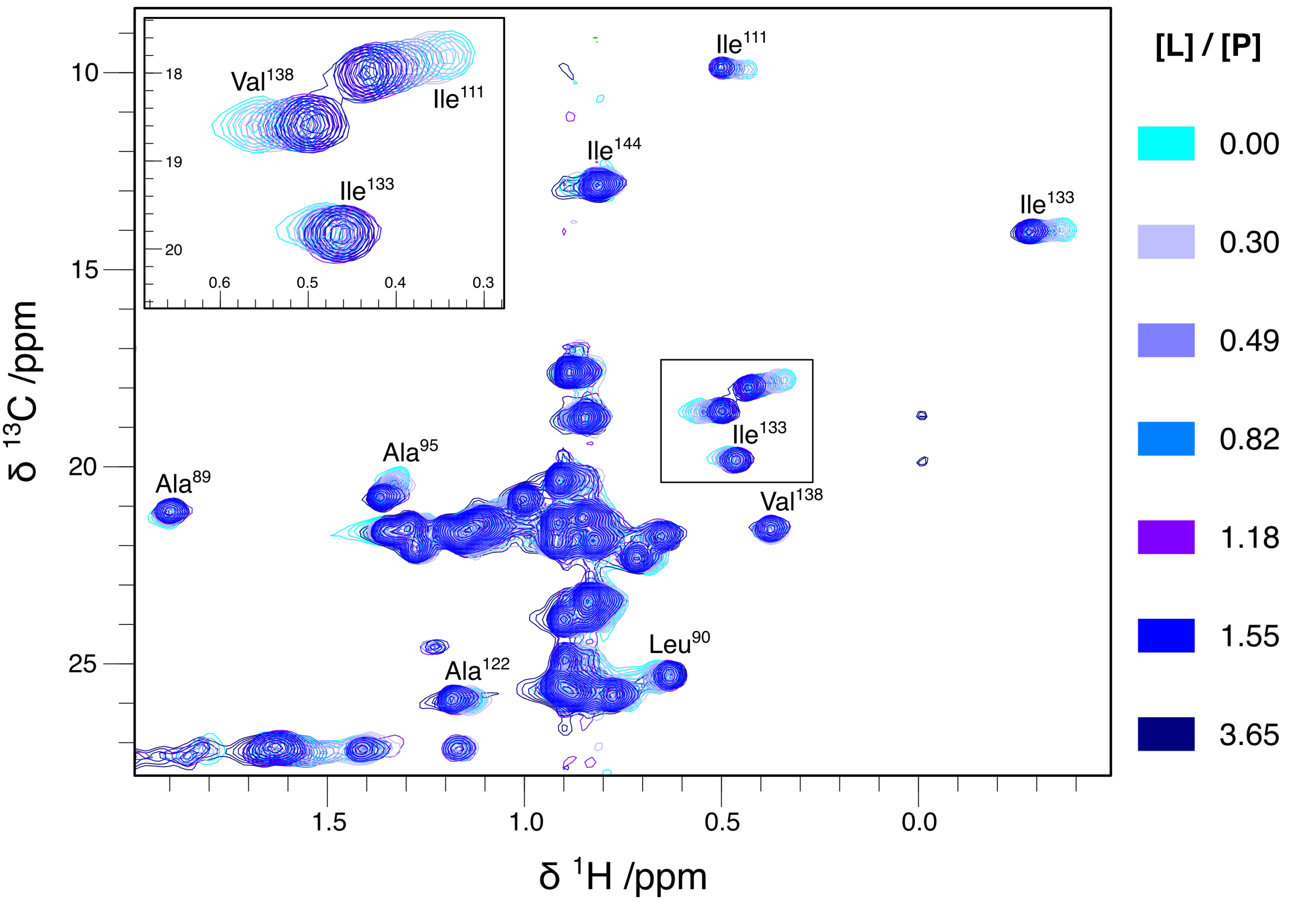

Figure S6: Overlay of selected ^{1}H-^{13}C HSQC spectra of hFynSH3, obtained during the automated titration experiment with p85$\alpha_{\text{P91-T104}}$. Colouring scheme from light blue to navy corresponds to increasing concentration of the p85$\alpha_{\text{P91-T104}}$ ligand, molar ratios are indicated on the right-hand side. For clarity, only selection of the assigned methyl signals are highlighted. The insert in the top left corner represents the expansion that is boxed in the centre of the spectrum.

The acquisition and performance parameters of the acquired spectra are listed in table 1. Following the

initial recording of the ^{1}H-^{13}C HSQC, the acquisition of the ^{1}H-^{15}N HSQC was optimized for signal-to-noise ratio (SNR), which has shown approximately 10-fold enhancement, from 2.7 to 31.5. Conversely, the linewidth of the ^{1}H-^{13}C HSQC signals is 33 Hz compared to 50 Hz observed in the ^{1}H-^{15}N HSQC. This difference is likely due to the favourable relaxation properties of the protein methyl groups. The SNR and linewidth ($\nu_{1/2}$) parameters both influence the apparent spectral resolution (ε) calculated as:

$$\varepsilon = \frac{\nu_{1/2}}{\sqrt{SNR}}. \tag{1}$$

In consequence, the apparent resolution has increased two-fold for the ^{1}H-^{15}N HSQC, from 20 to 9 Hz, reflecting the higher SNR. This has allowed for reliable evaluation of the dissociation constant, K_D.

	^{1}H-^{13}C HSQC	^{1}H-^{15}N HSQC
Acquisition time / ms	136 (^{1}H) / 19 (^{13}C)	92 (^{1}H) / 16 (^{15}N)
NS	32	128
Protein /μg	43	61
SNR	2.7	31.5
Resolution	20 Hz	9 Hz

Table 1: Critical sensitivity and resolution parameters for the acquired HSQC spectra.

3 K_D Fitting

Two models were evaluated based on the CSPs recognised in the ^{1}H-^{15}N HSQC spectra. In both cases the individual CSPs were fitted collectively to a common K_D, and the peak intensity of the reference compound (TSP) was used to evaluate the molar ratio of ligand-to-protein in the analysed mixture. In the first model (1-parameter model) the intensities of the TSP signals measured for the protein-free (I_A) and ligand-saturated (I_B) solutions, as well as the total protein ($[P]_t$) and ligand ($[L]_t$) concentration values were used directly to calculate the normalized ligand concentration, α, at each step of the titration:

$$\alpha = \left(\frac{I_{TSP} - I_A}{I_B - I_A} [L]_t \right) / 2[P]_t. \tag{2}$$

Resulting α values along with the calculated fractional shifts ($\Delta\delta/\Delta\delta_{max}$) were the input parameters for:

$$\Delta\delta/\Delta\delta_{max} = 1/2 + \alpha + \beta_D - \sqrt{\alpha^2 + 2\alpha\beta_D + {\beta_D}^2 + 1/4 + \beta_D - \alpha}, \tag{3}$$

where the K_D was the global free parameter, while the $\Delta\delta_{max}$ was evaluated analytically for every single CSP signal.

In the second model, the intercept and the slope of the linear relationship between α and the TSP peak intensity I_{TSP} were treated as free parameters in addition to K_D (3-parameter model). This was done because the experimentally obtained value of I_A is very sensitive to the exact position of the injection boundary between solutions a and b in the reservoir capillary. Therefore, rather than determining the parameters I_A, I_B, and α_{max} experimentally, the linear relationship was recast as

$$\alpha = m I_{\mathrm{TSP}} + c, \tag{4}$$

and the quantities m and c were treated as free fitting parameters. As shown in Table 2, both procedures lead to similar values for K_D, with similar confidence intervals. However, the residual of the fit is significantly lower for the 3-parameter fit. This is reflected in the F-test that compares the ratio of the square residuals of the two fits, with

$$F = \frac{{\sigma_{1p}}^2}{{\sigma_{3p}}^2} \frac{n-3}{2}, \tag{5}$$

where ${\sigma_{1p}}^2$ and ${\sigma_{3p}}^2$ are the square residuals of the 1-parameter and the 3-parameter fits, respectively, and n is the number of fitted data points. This must be compared to the critical value $F^{0.05}_{n-3,n-1}$ for n data points and the rejection probability of 0.05. The results are given in Table 2, and show that the 3-parameter fit is statistically strongly justified, as F exceeds the critical value by nearly three orders of magnitude.

	1-Parameter Model	3-Parameter Model
Fit variables	K_D	K_D, m, c
n	87	
K_D /μM	34 ± 9	48 ± 9
m	6.6×10^{-8}	$(6.7 \pm 0.1) \times 10^{-8}$
c	-2.9×10^{-2}	$(4.1 \pm 0.5) \times 10^{-2}$
σ^2	0.48	0.02
F	966	
$F^{0.05}_{n-3,n-1}$	1.45	

Table 2: Comparison between the considered fitting models based on the CSP data.

References

[1] M. Plata, W. Hale, M. Sharma, J. M. Werner, and M. Utz. Microfluidic platform for serial mixing experiments with in operando nuclear magnetic resonance spectroscopy. *Lab Chip*, 21(8):1598–1603, Apr. 2021. ISSN 1473-0189. doi: 10.1039/D0LC01100B.

[2] M. Sharma and M. Utz. Modular transmission line probes for microfluidic nuclear magnetic resonance spectroscopy and imaging. *Journal of Magnetic Resonance*, 303:75–81, June 2019. ISSN 1090-7807. doi: 10.1016/j.jmr.2019.04.007.

[3] A. Yilmaz and M. Utz. Characterisation of oxygen permeation into a microfluidic device for cell culture by in situ NMR spectroscopy. *Lab Chip*, 16(11):2079–2085, May 2016. ISSN 1473-0189. doi: 10.1039/C6LC00396F.
